# The Phase Evolution and Physical Properties of Binary Copper Oxide Thin Films Prepared by Reactive Magnetron Sputtering

**DOI:** 10.3390/ma11071253

**Published:** 2018-07-20

**Authors:** Weifeng Zheng, Yue Chen, Xihong Peng, Kehua Zhong, Yingbin Lin, Zhigao Huang

**Affiliations:** 1 Fujian Provincial Key Laboratory of Quantum Manipulation and New Energy Materials, College of Physics and Energy, Fujian Normal University, Fuzhou 350117, China; wfzheng3@fjnu.edu.cn (W.Z.); jessica_p@126.com (X.P.); Khzhong@fjnu.edu.cn (K.Z.); Yblin@fjnu.edu.cn (Y.L.); 2 Fujian Provincial Engineering Technical Research Centre of Solar-Energy Conversion and Stored Energy, Fuzhou 350117, China; 3 Fujian Provincial Collaborative Innovation Center for Optoelectronic, Semiconductors and Efficient Devices, Xiamen 361005, China

**Keywords:** binary copper oxide, phase structure, band gap, contact potential difference

## Abstract

P-type binary copper oxide semiconductor films for various O_2_ flow rates and total pressures (*P*_t_) were prepared using the reactive magnetron sputtering method. Their morphologies and structures were detected by X-ray diffraction, Raman spectrometry, and SEM. A phase diagram with Cu_2_O, Cu_4_O_3_, CuO, and their mixture was established. Moreover, based on Kelvin Probe Force Microscopy (KPFM) and conductive AFM (C-AFM), by measuring the contact potential difference (*V*_CPD_) and the field emission property, the work function and the carrier concentration were obtained, which can be used to distinguish the different types of copper oxide states. The band gaps of the Cu_2_O, Cu_4_O_3_, and CuO thin films were observed to be (2.51 ± 0.02) eV, (1.65 ± 0.1) eV, and (1.42 ± 0.01) eV, respectively. The resistivities of Cu_2_O, Cu_4_O_3_, and CuO thin films are (3.7 ± 0.3) × 10^3^ Ω·cm, (1.1 ± 0.3) × 10^3^ Ω·cm, and (1.6 ± 6) × 10^1^ Ω·cm, respectively. All the measured results above are consistent.

## 1. Introduction

P-type binary copper oxide semiconductors with different morphologies and copper oxidation states have three distinct phases: cuprous oxide (Cu_2_O), paramelaconite (Cu_4_O_3_), and tenorite (CuO) [1,2]. They have great application potential in thin-film devices such as solar cell [3] and thin-film lithium-ion battery [2]. Many efforts have been made to further understand the thin film physical properties in theoretical calculations [1,4,5] and experiments [6,7,8,9]. The crystal symmetries of Cu_2_O, Cu_4_O_3_, and CuO vary from cubic to tetragonal and monoclinic, resulting in the diversity of optical and electronic properties.

The band structure of Cu_2_O, with a direct gap range from 2.1 to 2.6 eV [7,10,11,12], was experimentally well established. Although Cu_2_O has the advantage of good transparency in the visible light range, its low carrier concentration or large resistivity leads to poor performances [3,10]. The second oxide phase, Cu_4_O_3_, discovered during the late 1870s [13], is a metastable mixed-valence intermediate compound between Cu_2_O and CuO [1,4,9,14,15]. To date, research about the electronic structure of Cu_4_O_3_ has been limited. The estimated band gap by optical methods varies from 1.3 to 2.5 eV, depending on whether a direct or indirect gap was assumed for the analysis [4,14]. Recently, Wang et al. predicted that the indirect band gap of Cu_4_O_3_ is 1.59 eV [4]. As for CuO, the type of band gap of CuO remains controversial; in some studies its band gap is suggested to be direct [16,17,18], but it is considered that its band gap is indirect in other studies [1,19,20], and its accurate band gap value is still a greater challenge for electronic structure calculations.

Therefore, there is an urgent need to verify the calculated electronics structure of binary copper oxides through experiments. Various methods have been used to prepare binary copper oxides thin films. They include thermal oxidation [21,22], spray-coating [23], pulsed laser deposition [24,25], electrochemical deposition [26], and reactive sputtering [9,11,12,14]. Among those methods, magnetron sputtering at room temperature is desirable for the growth of thin films with good physical properties. Moreover, one can easily deposit the three types of binary copper oxides or their mixed phases by merely tuning the oxygen partial pressure during depositions [9,14,15].

The oxygen partial pressure during depositions does influence the oxygen chemical potential inside the deposition chamber. On one hand, the films deposited under lower oxygen partial pressure tend to form the Cu_2_O phase which contains only Cu^+^, and higher oxygen partial pressure will further oxidate Cu^+^ into Cu^2+^, resulting in the formation of the CuO phase. The calculated phase stability of the copper oxide system indicates that Cu_4_O_3_ is a metastable state [1,4], which means that the processing window of O_2_ flow to synthesize Cu_4_O_3_ is extremely narrow. Consequentially, the critical parameters for the synthesis of the Cu_4_O_3_ metastable phase need insightful exploration. On the other hand, the physical properties of thin films (such as preferred orientation, optical band gaps, mobilities, and carrier concentrations) can also be tuned by changing the oxygen partial pressure during deposition [9,14,15]. The effects during depositions of oxygen chemical potential on the films’ physical properties still need to be investigated further.

In this work, binary copper oxide thin films including Cu_2_O, Cu_4_O_3_, and CuO were prepared by DC magnetron sputtering under different oxygen partial pressures. The crystal structures of those binary copper oxide films were studied using XRD and Raman spectra; band gaps were measured by introducing a UV–vis spectrophotometer; and the nanoscale electrical property was investigated by conductive AFM (C-AFM). Additionally, the oxide states of Cu on the film’s surface were determined by Kelvin Probe Force Microscopy (KPFM). It is hoped that these experimental results can facilitate the better understanding of the thin film growth mechanism and the tuning effect of physics properties of binary copper oxide thin films.

## 2. Experiments

The binary copper oxide films were grown at room temperature by reactive magnetron sputtering. In the experiment, a Cu target of 2 inches with 99.999% purity was used. By using deionized water, acetone, and methanol, the glass substrates were rinsed ultrasonically. By blowing nitrogen gas, these substrates were dried in case of deposition. Then, the substrates were installed on a holder 10 cm away from the target. The rotation rate of 15 rpm was fixed during the deposition. The vacuum chamber was evacuated until the base pressure reached 4 × 10^−4^ Pa. The operating pressure varies from 0.5 to 2.0 Pa. Mixed argon–oxygen was used as the reactive gas. The oxygen flow rate was changed from 1 to 30 sccm, while the argon flow rate was kept at 50 sccm. A fixed DC power of 160 W was used for all the films. The deposition time was set to be 10 min. In order to measure the optical characterization, transparent glass substrates were used. However, in order to obtain *J*–*E* curves, a conductive substrate (ITO—indium tin oxide) was also considered.

The crystalline structures of the films were measured using XRD (RigakuMiniFlex II, Rigaku, Tokyo, Japan) with Cu Kα radiation of λ = 1.5418 Å and using Raman spectroscopy (HORIBA Jobin Yvon Evolution, Jobin Yvon, Paris, France). The scanning speed of XRD characterization was set to 5°/min in order to ensure sufficiently strong diffraction intensity. The surface morphologies were observed using SEM (Hitachi SU-8010, Tokyo, Japan). Based on Dektak XT (Bruker, Hamburg, Germany), the thickness of the films was obtained. Using a UV–vis spectrophotometer (Shimadzu UV-Vis 2450, Kyoto, Japan), we measured the optical reflectance and transmission spectra. The work functions and I–V curves were recorded using KPFM (Bruker Dimension Icon, Hamburg, Germany) and C-AFM measurements, respectively (AFM, Bruker Dimension Icon, Hamburg, Germany). In order to avoid the influence of moisture and gas absorption on the measured results, the whole AFM was put into a glove box with water and oxygen content <0.1 ppm. 

## 3. Results and Discussion

**Figure 1:**
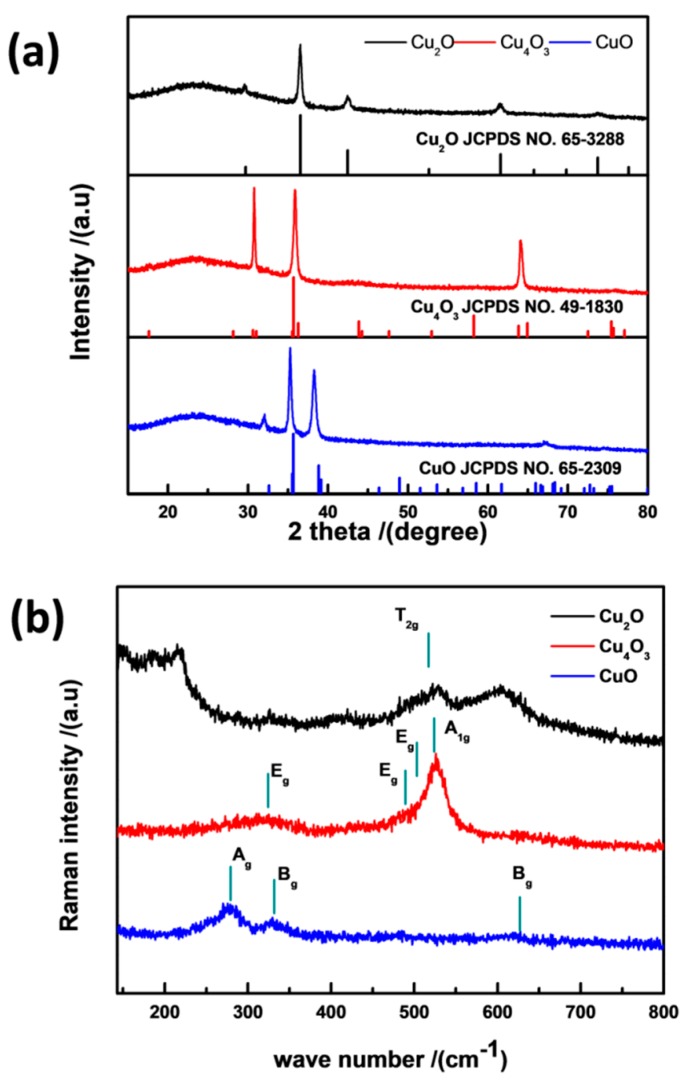
The XRD (**a**) and Raman spectra (**b**) of Cu_2_O, Cu_4_O_3_, and CuO deposited at 0.5 Pa with the flow rates of 8 sccm, 14 sccm, and 24 sccm, respectively.

Figure 1a shows the XRD patterns for pure phase Cu_2_O, Cu_4_O_3_, and CuO deposited at 0.5 Pa with the flowing rates of 8 sccm, 14 sccm, and 24 sccm, respectively. From the figure, one can notice that the peaks of the three samples are consistent with those characteristic of the cuprous oxide, paramelaconite, and tenorite phases (JCPDS NO. 65-2388, 49-1830, and 65-2309), respectively. All the observed diffraction peaks are summarized in Table 1. Raman spectra were also introduced to confirm the film structure and detect the trace impurity. As shown in Figure 1b, all the Raman peaks marked using vertical bars agree well with experiments [15] and with previous calculations [27]. The XRD and Raman results indicate that the three types of Cu_2_O, Cu_4_O_3_, and CuO films can be prepared through magnetron sputtering by only tuning the O_2_ flowing rate.

**Figure 2:**
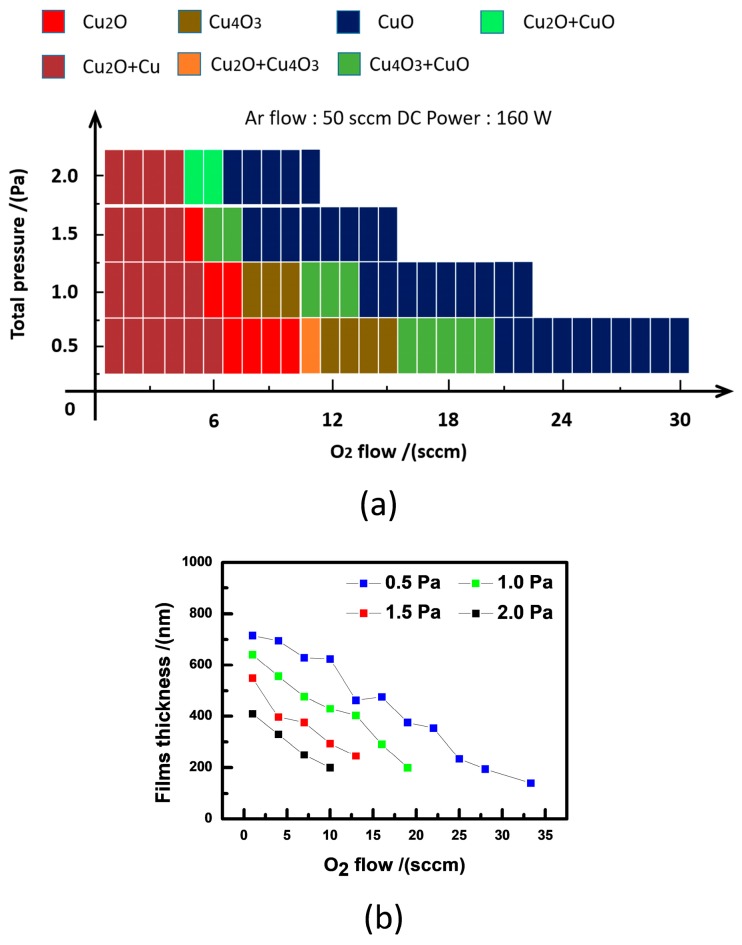
(**a**) The schematic deposition diagram of films deposited under different total pressure and O_2_ flow rate; (**b**) film thickness prepared under different conditions.

By using XRD and Raman measurements, the phase diagram of Cu_x_O_y_, deposited under different O_2_ flow rates and total pressures, is shown on Figure 2a. From the figure, we can see that the increase of the oxygen flowing rate at 0.5 Pa results in the evolution from pure Cu_2_O, to a mixture of Cu_2_O and Cu_4_O_3_, to pure Cu_4_O_3_, to a mixture of Cu_4_O_3_ and CuO, and to pure CuO. However, further increase of the O_2_ flow rate will give rise to the deterioration of the film crystallinity of CuO. This is consistent with previous results [9,14].

As the total pressure is enhanced to 1.0 Pa, the processing windows of O_2_ flow rate to synthesize a mixture of Cu_2_O and Cu_4_O_3_ disappeared, and the O_2_ flow processing window for pure-phase Cu_2_O and Cu_4_O_3_ became narrower. Moreover, the pure phase domains of Cu_2_O and Cu_4_O_3_ are moved to lower O_2_ flow rate magnitude, which indicates that, for the larger total pressure, a lower oxygen flow rate can produce copper oxide with higher valence. Additionally, it is worth mentioning that the pure Cu_4_O_3_ and Cu_2_O phases disappear at 1.5 Pa and 2.0 Pa, respectively. It is also found that the phases are represented by Cu_2_O, Cu_4_O_3_, CuO, Cu, and their mixtures, which means that grains of intermediate composition Cu_x_O_y_ may not present under such deposition conditions. As seen in Figure 2b, the film thicknesses deposited with 0.5 Pa, 1.0 Pa, 1.5 Pa, and 2.0 Pa under 1 sccm O_2_ flow rate were obtained at about 700 nm, 620 nm, 550 nm, and 400 nm, respectively. Moreover, the film thickness is reduced with increasing oxygen flowing rate for same total pressure, reducing with increasing total pressure for same O_2_ flowing rate.

At a fixed argon flow rate, the increase of the total pressure means that of the O_2_ partial pressure. The O_2_ partial pressure influences the morphology of deposited films.

**Figure 3:**
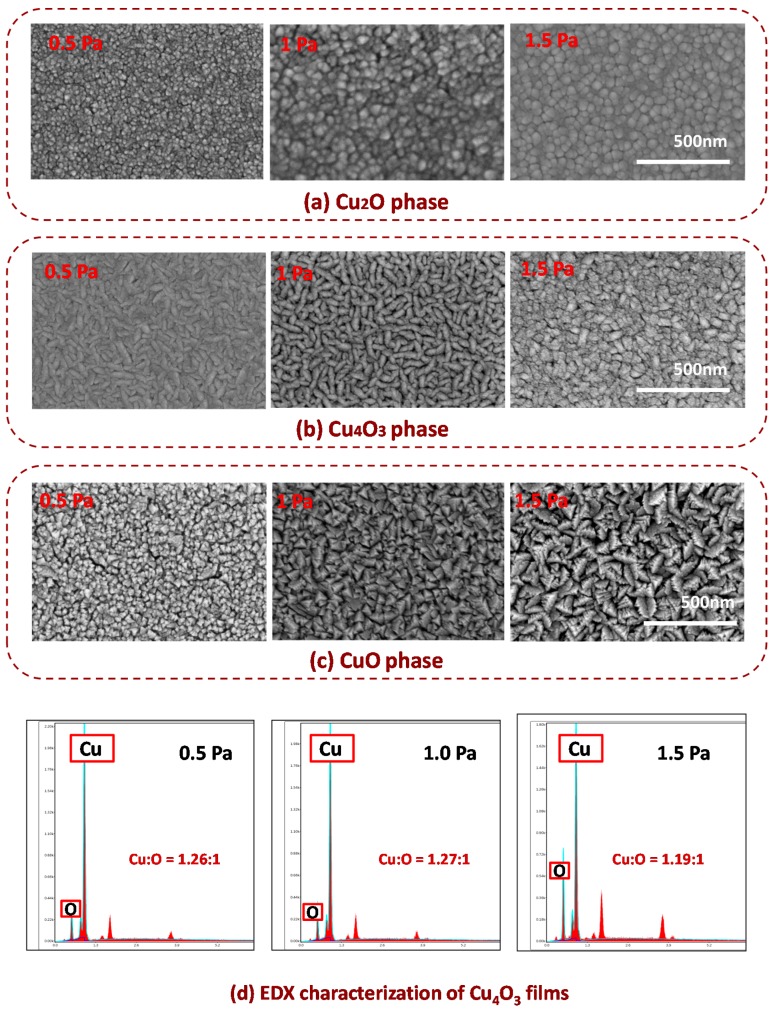
SEM images for the morphology evolution of the deposited films under various total pressures for (**a**) Cu_2_O; (**b**) Cu_4_O_3_, and (**c**) CuO; (**d**) the EDX characterization of Cu_4_O_3_ films deposited under 0.5 Pa, 1.0 Pa, and 1.5 Pa, respectively.

The evolution of the film morphologies under various total pressures is shown in Figure 3. From the figure, one notices that the surface roughness of the binary copper oxide increases with increasing oxygen partial pressure. The surface of the Cu_2_O thin film consists of a lot of “spherical” grains, while the Cu_4_O_3_ and CuO thin films consist of many “roof-type” and “pyramidal-shape” grains, respectively. Especially, the Cu_4_O_3_ thin films deposited under 1.5 Pa contain the CuO phase which forms many “pimples” on top of the Cu_4_O_3_ “roof”. As shown in Figure 3d, an EDX compositional analysis of Cu_4_O_3_ deposited at 0.5 Pa and 1.0 Pa indicates that Cu-to-O atomic ratios are 1.26:1 and 1.27:1, respectively, which is close to the stoichiometric ratio with 1.33:1. However, the Cu-to-O atomic ratio of deposited films at 1.5 Pa deviates from 1.26:1, which indicates that CuO phase may exist in the Cu_4_O_3_ films. In addition, the existence of a CuO impurity phase was also confirmed by the following optical band characterization. The morphology of pure-phase thin films is closely related to their crystal structure, which was discussed in detail in other studies [9]. From our measured results, it is suggested that binary copper oxide films with fine electrical quality should be prepared under lower total pressure.

**Figure 4:**
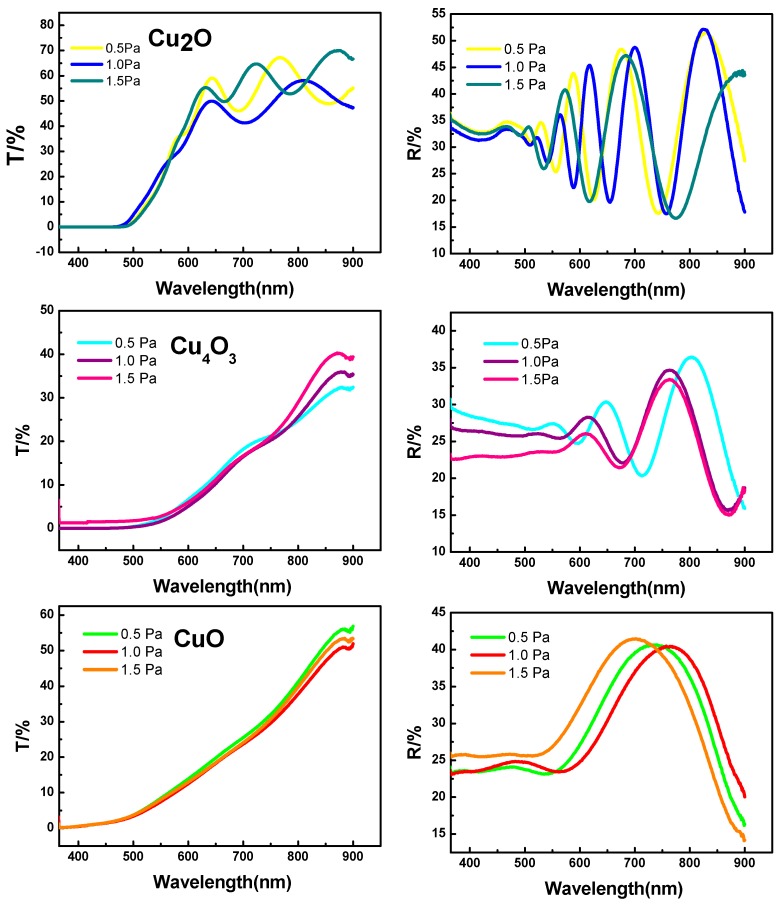
The transmittance and reflectance spectra of Cu_2_O, Cu_4_O_3_, and CuO thin films deposited under various total pressures.

The optical band gaps of Cu_2_O, Cu_4_O_3_, and CuO were also analyzed. The transmittance and reflectance spectra for different copper oxides deposited under various total pressures are present in Figure 4. By using the Tauc relation, one can estimate the *E_g_* values from the transmittance and reflectance [12,28]:

(1) $${\left(\alpha h\upsilon \right)}^{n}=A(h\upsilon -E_{g})\;$$

where *hν* is the incident photon energy, and *A* is a constant related to the materials. The magnitudes of *n* are considered to be 2, 1/2, 3, and 3/2 corresponding to allowed direct, allowed indirect, forbidden direct, and forbidden indirect transitions, respectively. 

Here, for CuO, the indirect band gap is considered, so *n* = 1/2. Moreover, Cu_2_O and Cu_4_O_3_ are supposed to a direct transition so *n* = 2 is considered [1,4,19]. The absorption coefficient *α* can be obtained based on following relation:

(2) $$\alpha =\frac{1}{d}\ln\left[\frac{{\left(1-R\right)}^{2}}{T}\right]$$

where *d* is the thickness of the film, and *R* and *T* are the reflectance and transmittance.

**Figure 5:**
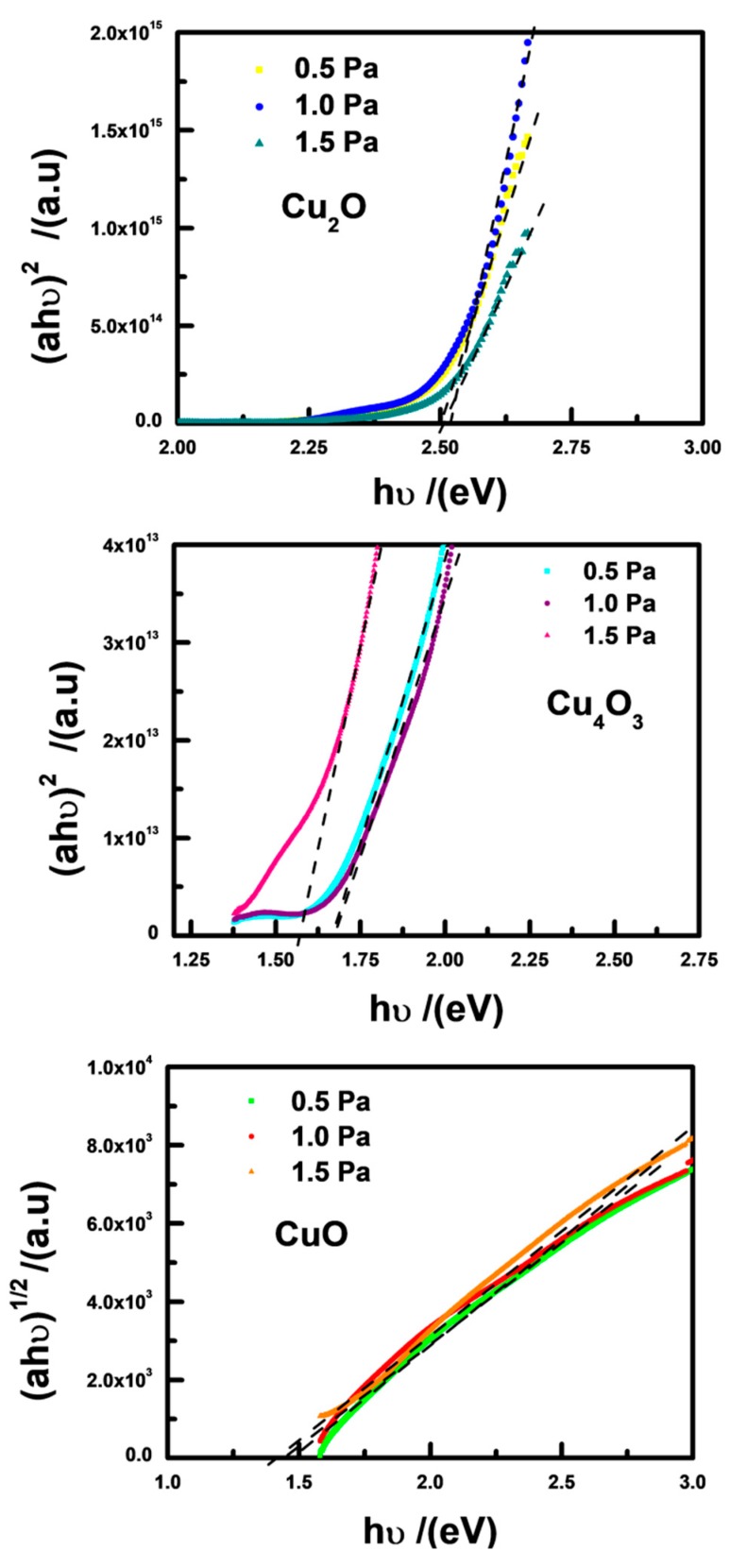
(***α****h**ν***)^n^ as a function of photon energy (*h**ν***) for pure-phase Cu_2_O, Cu_4_O_3_, and CuO thin films.

Figure 5 presents the photon energy dependence of the (***α****h**ν***)^n^ values. The calculated optical *E*_g_ values can be obtained as 2.51 ± 0.02 eV, 1.65 ± 0.1 eV, and 1.42 ± 0.01 eV for Cu_2_O, Cu_4_O_3_, and CuO, respectively. These are consistent with the previous reported results [2,4,7,12,15,28]. Furthermore, the measured results of the band gap indicate that, although the morphologies of the films under various O_2_ partial pressures are different, the band gap value of each type of single-phase copper oxide remains almost constant. This informs us that the band gap of binary copper oxide films can be tuned by controlling the ratio of Cu_2_O/Cu_4_O_3_/CuO in the mixed phase.

Compared with XPS, Raman, and FTIR with spatial resolution at the micrometer scale, the KPFM measured method allows us to distinguish between the Cu oxide states with nanometer resolution, and to observe the local morphology of thin films simultaneously [29]. There exists a contact potential difference (*V*_CPD_) between the scanning tip and the surface of sample; *V*_CPD_ can be described as follows [30]:

(3) $$V_{CPD}=\frac{\left(\varphi_{tip}-\varphi_{s}\right)}{q}\;$$

where *φ*_s_ is the work function of the sample, *φ*_tip_ is that of the tip, and *q* is the electronic charge. By measuring the work function of a standard sample (such as Au), the magnitude of *φ*_tip_ can be gained. Therefore, according to Equation (3), by measuring the value of *V*_CPD_, *φ*_s_ can be determined.

**Figure 6:**
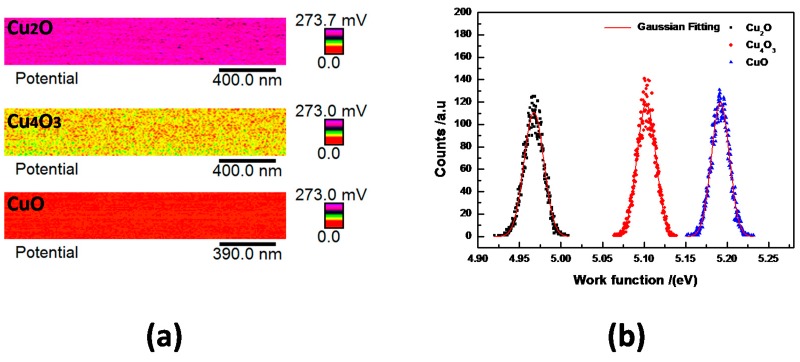
The *V*_CPD_ (**a**) and work function distribution (**b**) on the surface of Cu_2_O, Cu_4_O_3_, and CuO thin films.

Figure 6a,b present *V*_CPD_ and the work function distribution on the respective surfaces of Cu_2_O, Cu_4_O_3_, and CuO thin films. These data were obtained inside a 1 × 0.3 (μm)^2^ scanning region on the surface of the films, and the measured mean *V*_CPD_ values for Cu_2_O, Cu_4_O_3_, and CuO thin films are 231.0 mV, 98.5 mV, and 8.7 mV, respectively. According to Equation (3), the positive *V*_CPD_ values indicate that the work functions of the thin films are lower than the value of *φ*_tip_. The results indicate that the thin films of CuO and Cu_4_O_3_ containing Cu^2+^ have lower surface potential. From Figure 6b, it is found that $\phi_{Cu_{2}O}<\phi_{Cu_{4}O_{3}}<\phi_{CuO}$, which is consistent with other experimental results [29]. In addition, the copper oxide state can be identified with KPFM by a corresponding measurement *V*_CPD_ value range or work functions, and KPFM facilitates the undamaged characterization of the Cu oxidation state on binary copper oxide thin film surfaces, which should have wide application prospects. 

**Figure 7:**
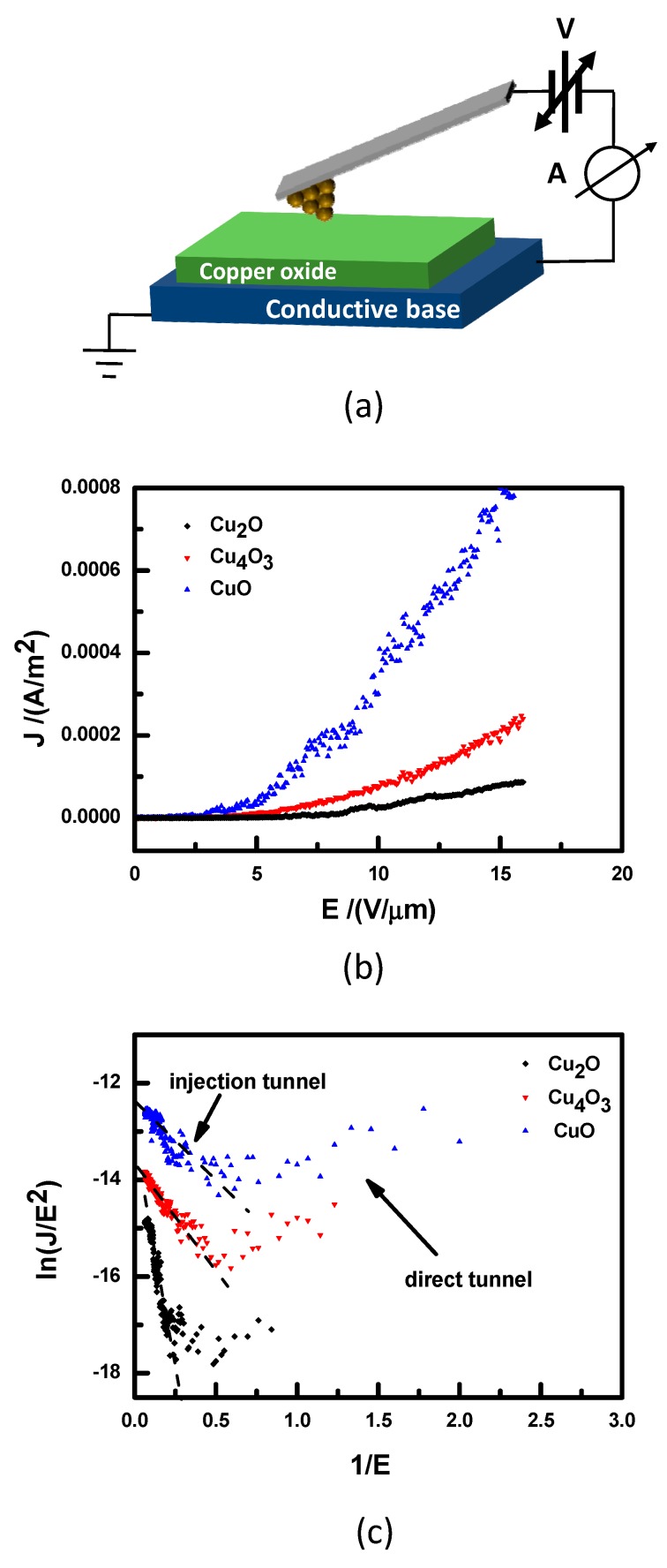
(**a**) A sketch of the C-AFM measurement system; (**b**) *J–E* curves of field emission; (**c**) ln(*J/E*^2^) versus 1/*E* plots of samples 1–4.

To further study the electronic properties of the binary copper oxide thin films, we used the C-AFM measurement system, as seen in Figure 7a. Here, a conductive tip (*R*_c_ ≈ 20 nm and *k* = 2.8 N/m) was used and a constant force (150 nN) was applied. This is similar to a tip-to-sample space mold in measuring *J*–*E* [31]. 

Our studied Tip–CuO–base should belong to the metal–insulator–metal (MIM) system. For this MIM case, a nonresonant tunnel transport has been established [31,32]. There exists a metal–insulator contact barrier φ produced by the insulator in MIM. Now, a bias voltage *V* is applied to the MIM system. Then, as the value of φ is less than *qV*, an injection tunnel current will be produced. However, as φ > *qV*, a direct current will arise. In order to analyze the properties of the field emission, the following *F–N* equation is generally used [31,32,33,34]:

(4) $$J=\frac{A\;\beta^{2}E^{2}}{\phi_{s}}\;\exp(\frac{-B\varphi_{}{}^{3/2}}{\beta E})\;$$

Equation (4) can be rewritten as the following:

(5) $$\ln(\frac{J}{E^{2}})=\ln(\frac{A\;\beta^{2}}{\varphi_{s}})-\frac{B\varphi^{3/2}}{\beta }(\frac{1}{E})\;$$

where $\varphi \;=\;\varphi_{tip}-\varphi_{s}$; *E* is the applied electric field; *J* is the current density (*A*·cm^−2^); *β* is the field enhancement factor; and *A* and *B* are constants. 

Figure 7b,c present the *J–E* curves and their ln(*J*/*E*^2^) versus 1/*E* plots of Cu_2_O, Cu_4_O_3_, and CuO, respectively. Both clear direct and injection tunnel regimes can be found in the figure. As found in Equation (5), according to the slope of ln(*J*/*E*^2^) versus 1/*E* plots, the φ information can be acquired. From Figure 7c, we can see that the slope of ln(*J*/*E*^2^) versus 1/*E* plots in the injection region increases in the order of Cu_2_O, Cu_4_O_3_, and CuO, which means that the value of φ is reduced in this order. That is, $\phi_{Cu_{2}O}<\phi_{Cu_{4}O_{3}}<\phi_{CuO}$, which is consistent with the observed result in Figure 5b. Moreover, in the direct tunnel region, it is found that, compared with Cu_2_O film, the current density *J* is evidently enhanced for Cu_4_O_3_ and CuO thin films, which indicates that the carrier concentration at room temperature increases for thin films deposited under higher O_2_ partial pressure. The above result is related to the observed fact of the band gap in Figure 5.

Finally, the resistivities of Cu_2_O, Cu_4_O_3_, and CuO thin films measured by the four-point probe method are (3.7 ± 0.3) × 10^3^ Ω·cm, (1.1 ± 0.3) × 10^3^ Ω·cm, and (1.6 ± 0.6) × 10^1^ Ω·cm, respectively. Clearly,$\rho_{{\mathrm{Cu}}_{2}O}>\rho_{{\mathrm{Cu}}_{4}O_{3}}>\rho_{\mathrm{CuO}}$. The resistivity values of CuO thin films are nearly 2 magnitudes less than those of Cu_2_O and Cu_4_O_3_, which should be attributed to the higher intrinsic carrier density of CuO [28,35,36]. The measured result indicates that the Cu_2_O film with the largest resistivity has the largest band gap and the least Cu valence state, while the CuO film with the least resistivity has the smallest band gap and the largest Cu valence state. All the measured results above are consistent. 

**Figure 8:**
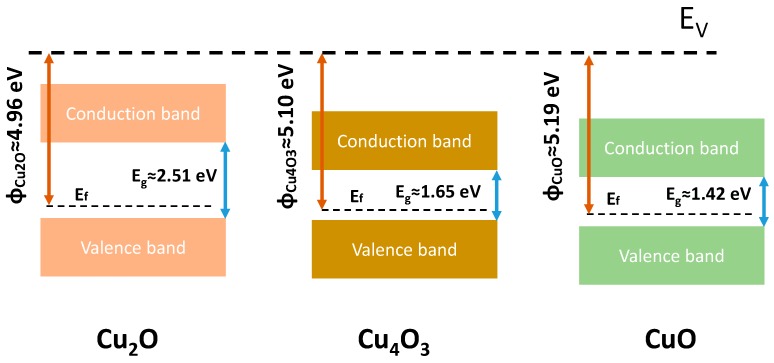
The band diagrams of Cu_2_O, Cu_4_O_3_, and CuO thin films.

The evolution of Cu valence states and the thickness of binary copper oxide films are typically affected by total pressure, O_2_ flow rate, substrate temperature, and so on. At room temperature, the interplay of total pressure and O_2_ flow rate leads to the complex change of the phase. The phase diagram and corresponding thickness change in Figure 2 should be associated with the deposition rate and energy of impinged atoms. For the same O_2_ flow rate, the low deposition rate for a high *P***_t_** value gives rise to the decrease in the incoming atom flow. High total pressure can reduce the contribution of the atomic bombardment, because the collisions of the sputtering atoms are enhanced. As a result, with increasing total pressure, the deposition thickness is decreased. Usually, a larger O_2_ flow rate can lead to higher energy of negative oxygen ions (O^−^) [37], which indicates that the bombardment effect on the deposition surface should be severer in binary copper oxide films with larger O_2_ flowing rate. Thus, for the same total pressure, with increasing O_2_ flow rate, the deposition thickness is decreased. However, on the other hand, higher energy of O^−^ under larger O_2_ flow rate can give rise to a more sufficient reaction between Cu^+^ and O^−^. As a consequence, at low O_2_ flow rate, Cu_2_O phase is mainly formed due to insufficient O_2_ and lower energy of O^−^. With increasing O_2_ flow rate, the reaction between Cu^+^ and O^−^ is gradually enhanced, which leads to some of Cu^+1^ being oxidized to become Cu^+2^. Thus, Cu_4_O_3_ phase (Cu_2_O + 2CuO) is formed. Similarly, a larger O_2_ flow rate can lead to all of Cu^+1^ being oxidized to Cu^+2^, which gives rise to the formation of pure CuO. Based on the measured results of the band gap and work function in Figure 5 and Figure 6, an illustration of the band diagrams of Cu_2_O, Cu_4_O_3_, and CuO films is presented in Figure 8. From the figure, it is found that the magnitudes of the band gap for Cu_2_O, Cu_4_O_3_, and CuO films are consistent with the other experimental results [38]. However, the experimental gap for Cu_2_O is in good agreement with that calculated based on hybrid functional calculations, while there are discrepancies between experiment and theory for CuO and Cu_4_O_3_ [1]. This may be associated with the defects in the prepared films, which need to be clarified by further experimental and theoretical investigations. 

The developments of film characterization techniques supply more tools to produce insight into the microscopic mechanism of physical properties for the films. Here, we introduced a nondestructive characterization approach, KPFM, to distinguish the surface electronic states depending on the composition. In general, the moisture, surface charge, absorption, and so on can evidently influence the measured accuracy of the work function [39,40,41]. Thus, in the measuring process, these adverse factors should be overcome. The direct valence measurement by X-ray photoelectron spectroscopy (XPS) can detect not only the information from the film’s surface, but also a depth of penetration. Therefore, the integration of KPFM with XPS may be a tremendously exciting endeavor.

## 4. Conclusions

The Cu_2_O, Cu_4_O_3_, and CuO films were prepared through magnetron sputtering by changing the O_2_ flowing rate and total pressure. The phase diagrams and morphologies of Cu_2_O, Cu_4_O_3_, CuO, and their mixtures were established by structural analysis using XRD, SEM, and Raman spectrometry. One notices that the binary copper oxide films with fine electrical quality should be prepared under lower total pressure. Moreover, the contact potential difference and the field emission property were measured by KPFM and conductive AFM(C-AFM). It is found that $\phi_{Cu_{2}O}<\phi_{Cu_{4}O_{3}}<\phi_{CuO}$. The band gaps of Cu_2_O, Cu_4_O_3_, and CuO thin films were observed to be 2.51 ± 0.02 eV, 1.65 ± 0.1 eV, and 1.42 ± 0.01 eV, respectively. The resistivity values of the Cu_2_O, Cu_4_O_3_, and CuO thin films are (3.7 ± 0.3) × 10^3^ Ω·cm, (1.1 ± 0.3) × 10^3^ Ω·cm, and (1.6 ± 0.6) × 10^1^ Ω·cm, respectively. Moreover, the measured results indicate that the Cu_2_O film with the largest resistivity has the largest band gap and the least Cu valence state, while the CuO film with the least resistivity has the smallest band gap and the largest Cu valence state. All the measured results above are consistent. 

## Figures and Tables

**Table 1:** The summary of diffraction peaks in XRD patterns.

Cu_2_O		Cu_4_O_3_		CuO	
2θ (°)	(h k l)	2θ (°)	(h k l)	2θ (°)	(h k l)
36.5	1 1 1	30.7/31.1	2 0 0/1 0 3	35.5/35.7	0 0 2/$\bar{1}11$
42.4	2 0 0	35.6/35.7/36.3	2 0 2/0 0 4/2 2 0	38.9/39.1	1 1 1/2 0 0
61.5	2 2 1	63.9/65.0	4 0 0/2 0 6	65.6	0 0 2
73.6	3 1 1				
